# Synthesis of Polyformate Esters of Vegetable Oils: Milkweed, Pennycress, and Soy

**DOI:** 10.1155/2016/3128604

**Published:** 2016-02-03

**Authors:** Rogers E. Harry-O'kuru, Girma Biresaw, Brent Tisserat, Roque Evangelista

**Affiliations:** ^1^Bio-Oils Research Unit, National Center for Agricultural Utilization Research, United States Department of Agriculture-Agricultural Research Service, 1815 N. University Street, Peoria, IL 61604, USA; ^2^Functional Food Research Unit, National Center for Agricultural Utilization Research, United States Department of Agriculture-Agricultural Research Service, 1815 N. University Street, Peoria, IL 61604, USA

## Abstract

In a previous study of the characteristics of acyl derivatives of polyhydroxy milkweed oil (PHMWO), it was observed that the densities and viscosities of the respective derivatives decreased with increased chain length of the substituent acyl group. Thus from the polyhydroxy starting material, attenuation in viscosity of the derivatives relative to PHMWO was found in the order: PHMWO ≫ PAcMWE ≫ PBuMWE ≫ PPMWE (2332 : 1733 : 926.2 : 489.4 cSt, resp., at 40°C), where PAcMWE, PBuMWE, and PPMWE were the polyacetyl, polybutyroyl, and polypentanoyl ester derivatives, respectively. In an analogous manner, the densities also decreased as the chain length increased although not as precipitously compared to the viscosity drop. By inference, derivatives of vegetable oils with short chain length substituents on the triglyceride would be attractive in lubricant applications in view of their higher densities and possibly higher viscosity indices. Pursuant to this, we have explored the syntheses of formyl esters of three vegetable oils in order to examine the optimal density, viscosity, and related physical characteristics in relation to their suitability as lubricant candidates. In the absence of ready availability of formic anhydride, we opted to employ the epoxidized vegetable oils as substrates for formyl ester generation using glacial formic acid. The epoxy ring-opening process was smooth but was apparently followed by a simultaneous condensation reaction of the putative *α*-hydroxy formyl intermediate to yield vicinal diformyl esters from the oxirane. All three polyformyl esters milkweed, soy, and pennycress derivatives exhibited low coefficient of friction and a correspondingly much lower wear scar in the 4-ball antiwear test compared to the longer chain acyl analogues earlier studied.

## 1. Introduction

In this decade, use of vegetable oils in lubricant and fuel applications has witnessed increased interest. This presumably arose from the vacillating cost of petroleum-based fuels and lubricants. In particular, the nascent awakening of public awareness in health costs tied to environmental pollution being generated by the nonbiodegradability of petroleum-based products in the environment has stimulated this drive. Of course, knowing that both petroleum product prices and their pollution effects keep rising is one thing, but understanding that the raw material supply of these are nonrenewable is a major driving force for interest in alternatives, that is, what we can grow on the farm and control. Besides, when properly modified, products from renewable materials retain their inherent biodegradability after their useful application lifetime and therefore pose no threat to the environment. Polyesters of hydroxylated vegetable oils are readily made with acid anhydrides with traditional base catalysis. But whereas anhydrides of fatty acids of chain length (C2–C18) are readily available commercially, formic anhydride is not. So instead of generating formic anhydride for a reagent, we opted to modify the vegetable oil to the polyoxiranes that are expected to smoothly ring-open with available formic acid to give the target formate ester derivatives of the oils under moderate reaction conditions. Synthesis of the polyoxiranes was achieved in excellent yields from vegetable oils using known chemical procedures [1–4]. From the low pH 2.3 of formic acid, we had expected a facile epoxy ring-opening without a stronger mineral acid catalyst to generate the *α*-hydroxy formate esters. What was actually observed under gentle reflux was contrary to expectation, a vicinal diformyl ester of each oxirane moiety. The polyformyl* esters* were characterized by FT-IR, proton, and carbon NMR spectrometry. The derivatives were evaluated from measurements of their density, viscosity, coefficient of friction, and antiwear properties. The polyformyl triglyceride derivatives exhibited suitable characteristics as good lubricant agents.

## 2. Materials and Methods

### 2.1. Materials and Chemicals

Milkweed epoxy oil (EMWO) was prepared from literature methods using our in-house source of milkweed oil and soybean oil was purchased from a local grocery store in Peoria, IL, and used as obtained, whereas pennycress (*Thlaspi arvense* L.) oil was a hexane extract (Soxhlet) of press cake from a pennycress seed crush; the extracted oil was purified before use. Sodium chloride, sodium bicarbonate, sodium carbonate, ethyl acetate, hexane, and formic acid (99 + %) were purchased from ACRŌS ORGANICS (Vineland, NJ, USA). Hydrogen peroxide (50%) was purchased from Sigma Aldrich (St. Louis, MO, USA). Isopropyl alcohol (Certified ACS Plus) and hexanes (HPLC grade) used for cleaning specimens and instruments for tribological investigations were purchased from Fisher Scientific (Pittsburgh, PA, USA). Water used in surface tension experiments was obtained by purifying deionized water to a conductivity of 18.3 megohms-cm on a UV/UF water purification system (EASYpure UV/UF, Model #: D8611, Barnstead International, Dubuque, IA, USA), followed by filtration on a 0.22 *μ*L sterile disposable filter (MILLEX-GS 0.22 *μ*L Filter Unit; Millipore Corporation, Bedford, MA, USA). All reagents were reagent grade materials and used as supplied.

Test balls used in the four-ball antiwear (4-Ball AW) experiments were obtained from Falex Corporation (Aurora, IL, USA) and had the following specifications: chrome-steel alloy made from AISI E52100 standard steel; 64–66 R_c_ hardness; 12.7 mm diameter; grade 25 extra polish. The test balls were degreased by two consecutive five min sonications in isopropyl alcohol and hexane solvents in an ultrasonic bath before use in the 4-ball AW experiments.

## 3. Methods

### 3.1. Instrumentation

#### 3.1.1. Fourier Transform Infrared (FT-IR) Spectrometry

FT-IR spectra were measured on an Arid Zone FT-IR spectrometer (ABB MB-Series, Houston, TX, USA) equipped with a DTGS detector. Liquid derivatives were pressed between two NaCl discs (25 mm × 5 mm) to give thin transparent oil films for analysis by FT-IR spectrometry. Absorbance spectra were acquired at 4 cm^−1^ resolution and signal-averaged over 32 scans. Interferograms were Fourier transformed using cosine apodization for optimum linear response. Spectra were baseline corrected, scaled for mass differences, and normalized to the methylene peak at 2927 cm^−1^ *NMR Spectroscopy* ^1^H and ^13^CNMR spectra were acquired on a Bruker AV-500 MHz Spectrometer with a dual 5 mm proton/carbon probe (Bruker, Billerica, MA, USA). The internal standard used was tetramethylsilane.

#### 3.1.2. Densities

Density measurements were performed using a pycnometer or specific gravity bottle Kimble Glass Inc. (capacity 10 mL) equipped with a thermometer (Kimble Glass Inc. Vineland, NJ, USA). The pycnometer was cleaned with acetone and dried with an air jet at room temperature. The components were reassembled and weighed empty. Each sample was carefully introduced into the bottle until the liquid meniscus almost grazed the lower portion of the ground glass joint. The thermometer (lid) was carefully inserted allowing any excess sample and air bubbles to exit through the open side arm. The completely filled pycnometer was carefully cleaned of excess sample, the side arm cap replaced, and the system was then weighed at the operating RT. The difference in mass from the empty pycnometer mass is the mass of 10.0 mL of sample. Two measurements for each sample were averaged to obtain the mass of each sample. Between samples, the pycnometer assembly was thoroughly rinsed with a mixture of hexane-acetone and dried before reuse.

#### 3.1.3. Viscosity Measurements

Measurements were determined on a Temp-Trol constant temperature bath (Precision Scientific, Chicago IL, USA) using Cannon-Fenske viscometer for transparent liquids (Cannon-Fenske Instrument Company, State College, PA, USA) in accordance with the American Oil Chemists (AOCS) Official Method Tq 1a-64 [5]. The size of the Cannon-Fenske viscometer used varied in accordance with the character of the oil derivative in order to stay within the range of the particular tube. The cleaned dry tube was loaded at RT with the sample oil and placed in its holder in the constant temperature bath. The sample was allowed to equilibrate for 10 min at 40°C or 15 min at 100°C before the sample was suctioned into the lower bulb until the meniscus just overshot the mark above the lower bulb. The suction was removed and the meniscus adjusted to the mark. The sample was allowed to flow at the same time the stop clock was started. The time (in seconds) it took the meniscus to reach the mark below the bulb multiplied by the tube constant gave the viscosity of the sample. The measurements were replicated for reproducibility. All kinematic viscosity measurements were run in duplicate and the average values were reported [6].

#### 3.1.4. Viscosity Index (VI)

Viscosity indices were calculated from kinematic viscosity data at 40 and 100°C following the procedure outlined in ASTM D 2270-93 [7] measurement system (Koehler Instrument Company, Inc., Bohemia, New York, USA).

#### 3.1.5. Surface Tension Measurements

Surface tension (also referred to as the equilibrium surface tension) of the oils was obtained from dynamic surface tension (surface tension vs. time) data measured using the axisymmetric drop shape analysis (ADSA) method [8]. In the ADSA method, surface tension is obtained by analyzing the change in the shape of a pendant drop of the oil suspended in air as a function of time. Dynamic surface tension was measured on the FTA 200 automated goniometer (First Ten Angstroms, Portsmouth, VA, USA). A detailed description of the instrument has been given elsewhere [9]. The main features of the instrument include an automated pump that can be fitted with various sizes of syringes and needles to allow for the control of the pendant drop formation; an automated image viewing and capturing system; software for automated drop shape analysis and surface tension measurement; computer hardware and software for data capture, storage, analysis, and transfer. All dynamic surface tension measurements were conducted at room temperature (23 ± 2°C). In a typical procedure, the oil was transferred into a 10 mL disposable syringe (Becton Dickinson & Co., Franklin Lakes, NJ, USA), equipped with a 17 gauge (1.499 mm OD) blunt disposable needle (KDS 17-1P, Kahnetics Dispensing Systems, Bloomington, CA, USA). The instrument was then programmed to automatically deliver a predetermined volume of the oil at 1 *μ*L/sec and also to automatically trigger image capture when the pump stops. The volume of pumped oil was selected so as to generate the largest possible pendant drop that will not fall off before image acquisition is completed. At the end of the acquisition period, each image was automatically analyzed and a plot of time vs. surface tension automatically displayed. The data from each run was saved as both a spreadsheet and a movie. The spreadsheet contained the time vs. surface tension data for each image and the movie contained each of the drop images as well as calibration information. Repeat dynamic surface tension measurements were conducted on each sample. Prior to running tests with the oils, the instrument was calibrated with purified water and then checked by measuring the interfacial tension between purified water and pure hexadecane. A dynamic surface tension data set shows the surface tension initially decreasing sharply with time, but leveling off to a more or less constant value at longer times. The (equilibrium) surface tension of an oil is determined from such data by averaging the values at very long times, where the surface tension shows little or no change with time.

#### 3.1.6. Pressurized Differential Scanning Calorimetry (PDSC)

PDSC tests were conducted according to ASTM D 6186-98 [10] on a Q20P Pressure Differential Scanning Calorimeter (TA Instruments-Waters LLC, New Castle, DE, USA). The instrument is fitted with a computer and software to allow for automatic data acquisition, for data analysis, and for determination of onset (OT) and peak temperatures (PT). All tests were conducted with the cell pressurized with pure oxygen to 500 ± 25 psig and a positive oxygen flow rate of 100 ± 10 mL/min. In a typical test, 1.0–2.0 mg sample of oil was placed into an aluminum pan which was then hermetically sealed; the top cover has a pinhole, and the pan is placed in the instrument cell. The cell is pressurized to 500 ± 25 psig, the temperature equilibrated at 50°C, and the oxygen flow rate adjusted to 100 ± 10 mL/min. The cell temperature is then ramped to 250°C at a rate of 10°C/min and the heat flow vs. temperature (and vs. time) data automatically recorded by the instrument computer. Duplicate PDSC runs were conducted on each sample, and average OT and PT values are reported.

#### 3.1.7. Four-Ball Tribometry

*Instrument and Specimen*. Four-ball tests were conducted on a model KTR 30 L 4-ball tribometer (Koehler Instruments, Bohemia, NY, USA). The instrument comprises a mechanical unit, an electronic unit, and a computer with TriboDATA software (Koehler Instruments, Bohemia, NY, USA) that allows for setting and controlling test parameters as well as for automatic data acquisition at a rate of 6–300 samples/min. The instrument specification is as follows: speed, 300–2000 rpm; maximum load, 10,000 N; maximum frictional torque, 20 Nm; temperature, ambient to 200°C. Standard test balls used in the 4-ball tests were obtained from Falex Corporation (Aurora, IL, USA) and had the following specifications: material, chrome-steel alloy made from AISI E52100 standard steel; hardness, 64–66 R_c_; diameter, 12.7 mm; finish, grade 25 extra polish. The balls were degreased by two consecutive sonications in isopropyl alcohol and hexane solvents in an ultrasonic bath before use. The pot and spindle used for securing the ball were also thoroughly washed with isopropyl alcohol and hexane, wiped with a Kimwipe® (Kimberly-Clark Worldwide, Inc., Roswell, GA, USA) and allowed to dry before use in 4-ball experiments.

#### 3.1.8. Four-Ball Antiwear (AW) Test

Four-ball antiwear (AW) tests were conducted following the procedure outlined in ASTM D 4172-94 method [11] under the following test conditions: load, 392 N; speed, 1200 rpm; lubricant temperature, 75°C; test duration, 60 min. During the test, the instrument computer automatically recorded test conditions (load, speed, and temperature) and frictional torque as a function of time at a rate of up to 300 samples/min. The coefficient of friction (COF) for each test was calculated from the torque and load data using the procedure outlined in ASTM D 5183 [12]. At the end of each test, the wear scar diameters (WSD) were measured in accordance with the procedure described in ASTM D4172 [11] using a wear scar measurement system supplied by Koehler Instrument Company, Inc. (Bohemia, NY, USA). The wear scar measurement system comprises hardware (for taking a digital image of the wear scar of the three balls without disassembling the ball pot) and software (ScarView, Koehler Instrument Company, Inc., Bohemia, NY, USA) for measurement of the WSD from the analysis of the digital images. Each test lubricant was used in at least two AW measurements and average COF and WSD values are reported.

#### 3.1.9. Four-Ball Extreme Pressure (EP) Test

The 4-ball EP test was conducted following the procedure outlined in ASTM D 2783 [13] under the following conditions: speed, 1760 ± 40 rpm; temperature, ambient; load, variable; test duration, 10 s. The EP test comprises a series of tests that are conducted at increasing loads until welding of the four balls occurs. After each test that did not result in a weld, the ball pot is removed, the lubricant emptied, the balls are rinsed with solvent, and the WSD were measured in accordance with the ASTM D4172 [11] procedure using the ScarView wear scar measurement system (Koehler Instrument Company, Inc., Bohemia, NY, USA). The load is then increased to the next higher selected value and the test repeated with a new set of clean balls. The load increments are selected based on the expected WP, with finer load increments being used close to the WP. The load at which welding is observed is the WP and is a characteristic EP property of the test lubricant.

#### 3.1.10. Synthesis of Polyepoxy Milkweed Oil

Milkweed oil (366.0 g, 418 mmol, d^25°C^ = 0.913 g·mL^−1^) was placed in a three-necked 1,000 mL jacketed reactor flask equipped with an overhead stirrer and initially maintained at 40°C. The oil was vigorously stirred and formic acid (29.4 g, 639 mmol, or 0.3 equiv/C=C) was added in one portion followed with a slow addition of H_2_O_2_ (50%, 200 mL, 7.18 mol). At the end of peroxide addition, the temperature was raised to 70°C and the reaction progress was monitored in 30 min intervals for disappearance of the 3009 cm^−1^ and 1654 cm^−1^ bands of the olefin and formation of the 820–845 cm^−1^ doublet of the oxirane as observed by FT-IR spectrometry. The reaction was judged to be complete in 4 h when the olefinic moieties were consumed. Heating was discontinued and the mixture was allowed to cool to RT. The colorless sludge was transferred into a separatory funnel with ethyl acetate and allowed to separate into organic and aqueous phases. The aqueous portion was removed and discarded while the organic phase was treated with saturated NaCl (200 mL containing 50 mL of saturated Na_2_CO_3_). The separated organic layer was rewashed with more NaCl solution and dried over MgSO_4_. The dried organic solution was filtered and concentrated under reduced pressure at 57°C followed by further drying at the pump to yield 395.0 g (98.8%). FT-IR film on NaCl cm^−1^: 2929 vs, 2863 s, 1743 vs, 1462 m-s, 1383 m, 1239 m, 1160 s, 843–824 d (asymmetric epoxy stretch), 722 m (-CH_2_- wag). ^1^HNMR (CDCl_3_) *δ*: 5.1 (m residual olefin), 4.18, 4.08 (-CH_2_-O-), 2.98, 2.85 (-H-C-O-C-H), 2.2 t (*J* = 7.5 Hz), 1.6 m, 1.5 bs, 1.4 bs, 1.2 bm, 0.85 m (9H).

^13^CNMR (CDCl_3_) *δ*: 172.88, 172.84, 172.45 (ester -C=O), 68.81 (-H-C-O- glyceryl), 61.91 (-CH_2_-O- glyceryl), 56.91, 56.86, 56.72, 56.67, 56.45, 56.39, 56.25, 54.10, 54.08, 53.94 (oxirane carbons), 33.94, 33.84, 33.82, 33.78, 31.78, 31.71, 31.63, 31.53, 29.55, 29.51, 29.47, 29.39, 29.35, 29.32, 29.20, 29.19, 29.18, 29.14, 29.13, 29.07, 29.02, 29.00, 28.94, 28.92, 28.88, 28.82, 28.78, 27.74, 27.69, 27.61, 27.24, 27.07, 26.79, 26.47, 26.44, 26.41, 26.31, 26.29, 26.12, 26.00, 24.70, 24.68, 24.64, 22.54, 22.51, 22.42, 13.96, 13.95, 13.91, 13.84.

#### 3.1.11. Synthesis of Milkweed Polyformate Esters

In a typical experiment, milkweed epoxy oil (215.20 g, 0.225 mol) prepared and characterized as in 3.1.10 [1–4] was placed in a 1 L dry round bottomed reaction flask containing a magnetic stir bar. Into this RBF was added 124.35 g (2.702 mol, 102 mL) formic acid and stirred. The reaction mixture was then heated to gentle reflux and monitored at 40 min intervals by sampling the contents for progress in the disappearance of the epoxy band at 820–840 cm^−1^ using FT-IR spectrometry. The reflux was allowed to run overnight after which FT-IR indicated complete disappearance of the oxirane absorption band. The heat source was then removed and the system allowed to cool to RT after which the mixture was transferred into a beaker of saturated NaHCO_3_ to neutralize excess formic acid. The solution was stirred and more NaHCO_3_ added until effervescence ceased. The organic phase was separated and saved while the aqueous layer was extracted twice with ethyl acetate (100 mL × 2). The combined organic phase was dried over Na_2_SO_4_ and concentrated under reduced pressure at 56°C followed by further drying with a vacuum pump to yield 294.6 g (98%) of the polyformyl ester of MWO. The product density was 1.09 g·mL^−1^ (24°C); viscosity 388.46 cSt (40°C) and 44.60 cSt (100°C), that is, a calculated VI = 172. The second derivative of the FT-IR spectrum (film on NaCl disc) *ν*_NaCl_ cm^−1^: 2958 s (-CH_3_ asym stretch), 2927 s (-CH_2_- asym stretch), 2875 s (-CH_3_ sym stretch), 2857 vs (-CH_2_- sym stretch), 1748 s (-C=O triglyceride ester), 1727 vs (-C=O formyl ester), 1469 m (-CH_2_- deform.), 1380 m-w (-CH_3_ deform), 1170 vs (-C-C-O- stretch), 724 w (-CH_2_- wag). ^1^HNMR (CDCl_3_) *δ*(ppm): 8.05–8.2 (s methine H-CO_2_, 10H), 5.18 (methine H-C-O glyceride backbone), 4.4–4.0 (-CH_2_O- glyceride backbone), 2.3 m, 2.05 s, 1.5 m, 1.3 m, 0.9 m (9H); ^13^CNMR (CDCl_3_) *δ*: 178.83, 171.16 (C=O triglyceride backbone); 161.01, 160.43, 160.30, 160.26 (-H-C=O formyl esters); 81.75, 81.67, 81.09, 80.99, 77.90, 77.79, 77.74, 77.39, 77.26, 74.91, 74.85, 74.80, 73.89, 73.29, 73.24 (-HC-O-); 68.87, 68.57, 68.49 (-CH-O- glyceride backbone); 62.07, 61.90, 61.82, 61.67, 61.42 (-CH_2_-O- glyceride backbone), 35.50, 35.42, 34.08, 34.01, 33.83, 31.89, 31.76, 31.52, 30.51, 29.62, 29.55, 29.40, 29.32, 28.89, 28.85, 28.65, 26.03, 25.78, 25.72, 25.10, 25.01, 24.93, 24.83, 24.68, 24.64, 24.56, 22.65, 22.59, 22.46, 22.43, 22.41, 22.35 (-CH_2_-); 20.99, 14.16, 14.08, 14.05, 13.97, 13.95, 13.92 (-CH_3_).

#### 3.1.12. Synthesis of Polyepoxy Soybean Oil

Into a dry three-necked 1.0 L jacketed reactor flask fitted with an overhead stirrer was placed 528.0 g of soybean oil. The contents of the flask were stirred vigorously while the temperature was increased to 40°C. At this temperature, formic acid (99 + %, 48.8 g, 40.0 mL) was added in one portion followed by dropwise addition of H_2_O_2_ (50%, 280.0 mL). At the end of H_2_O_2_ addition, the reaction temperature was raised to 70°C and progress of the reaction was monitored at 30 min intervals by sampling the reaction mixture followed by FT-IR analysis for the disappearance of the 3010 cm^−1^ band of the alkene and appearance of the 820–840 cm^−1^ of the oxirane doublet. The reaction was shown to be complete in 2 h. The heat source was removed and the product mixture allowed to cool to RT after which it was poured into saturated NaHCO_3_ to neutralize the excess formic acid. The product was extracted with ethyl acetate and washed with 50 mL saturated Na_2_CO_3_ in 200 mL of brine in a separatory funnel and the organic phase dried over anhydrous Na_2_SO_4_. The solvent was removed under reduced pressure at 57°C by rotary evaporation followed with additional drying with a vacuum pump to give 589.5 g (quantitative) of the oxirane. FT-IR *ν*_KBr_ cm^−1^: 2928 vs, 2856 vs, 1744 vs, 1463 s, 1383 m, 1242 m, 1159 s, 1106 m, 1018 m, 821 d, 711 m. ^1^HNMR (CDCl_3_) *δ* (ppm): 5.2 m (H-C-O- glyceride 1H), 4.25 m (4H), 4.1 m (CH_2_O- glyceride 2H); 3.09 m (4H), 2.95 bs (3H), 2.85 bs (epoxy 1H), 2.29 bs (6H), 1.72 m (4H), 1.58 bs (7H), 1.45 bs (18H), 1.3 m (42H), 0.86 m (9H). ^13^CNMR (CDCl_3_) *δ* (ppm): 173.17, 172.7 (-C=O triglyceride ester), 68.88 (methine of triglyceride), 62.03 (CH_2_O of glycerol); 57.11, 57.05, 56.92, 56.85, 56.64, 56.56, 54.25, 54.10 (oxirane); 34.07, 33.98, 33.91, 31.87, 31.80, 31.62, 29.63, 29.60, 29.56, 29.48, 29.41, 29.29, 29.24, 29.21, 29.16, 29.13, 29.12, 29.05, 28.93, 27.85, 27.84, 27.79, 27.76 (methylenes); 14.12, 14.10, 13.99 (terminal methyl groups).

#### 3.1.13. Synthesis of Polyformyl Ester of Soybean Oil

Soybean epoxide (440.0 g, 0.460 mol) from the above reaction was placed in a dry 1000 mL RBF containing a magnetic stir bar. Formic acid (99 + %, 150 mL, 3.9765 mol) was then added and the reaction flask was fitted with a Dean Stark and a reflux condenser and heated to gentle reflux while the mixture was stirred. As the reaction progressed, condensed water accumulated in the trap and the reaction was also monitored by sampling the mixture for diminution of the 825–845 cm^−1^ band of the starting oxirane. After 3.5 h, the FT-IR spectrum of a sample indicated the complete absence of the IR band of the epoxide. The heat source was removed and the reaction mixture allowed to cool to RT. The solution was poured into stirring saturated NaHCO_3_ solution and extracted with ethyl acetate (150 mL). The aqueous layer was further extracted with more ethyl acetate (100 mL × 2) and the combined organic phases dried over Na_2_SO_4_ and concentrated under reduced pressure at 57°C. The concentrate was pump-dried for 30 min to give 519.60 g of product, that is, 85.3% of theoretical; d^24°C^ = 1.041 g·mL^−1^. ^13^CNMR (CDCl_3_) *δ*: 171.16 (-C=O glyceride ester); 161.01, 160.43, 160.30, 160.27 (H-C=O ester); 128.99, 128.18 (residual olefin); 76.80, 73.28, 73.24 (oxygenated carbons), 68.90 (methine carbon of glycerol); 62.07, 61.67, 60.37 (methyleneoxy of glycerol), 35.41, 34.07, 34.01, 33.91, 33.87, 33.79, 31.88, 31.76, 31.50, 30.61, 30.50, 30.35, 29.65, 29.61, 29.58, 29.55, 29.43, 29.40, 29.31, 29.29, 29.26, 29.22, 29.11, 29.07, 29.05, 29.02, 28.95, 28.92, 28.88, 25.72, 25.01, 24.97, 24.93, 24.82, 24.69, 24.56, 22.64, 22.59, 22.46, 22.42, 22.41, 22.32, 20.99; 14.15, 14.07, 14.04, 13.94, 13.91, 13.87 (-CH_3_).

#### 3.1.14. Synthesis of Polyepoxy Pennycress Oil

Pennycress oil (298 g, 0.334 mol [14], d^25^ = 0.896 g·mL^−1^) was placed in a 1000 mL baffled, jacketed reaction flask equipped with an overhead stirrer and initially maintained at 40°C. To the stirred oil was added formic acid (99 + %, 20 mL) in one portion followed with a dropwise addition of H_2_O_2_ (50%, 150 mL). At the end of the H_2_O_2_ addition, the reaction temperature was increased to 70°C and epoxidation progress was monitored by sampling the reaction at 30 min intervals using FT-IR spectrometry as in the above soy epoxidation reaction. The heat source was removed after 1.5 h of reaction when the process was judged to be complete via FT-IR spectroscopy. Upon cooling to RT, the mixture was transferred into a saturated NaHCO_3_ solution to neutralize the excess formic acid. The product was extracted with ethyl acetate and washed with 50 mL saturated Na_2_CO_3_ in 150 mL saturated NaCl mixture and the organic phase dried over anhydrous Na_2_SO_4_. Removal of the solvent under reduced pressure at 57°C by rotary evaporation and further pump drying gave 290.3 g of the epoxy oil; d^25°C^ was 1.0231 g·mL^−1^; viscosity at 40°C was 136 cSt and 17.95 cSt at 100°C, respectively, which calculates to VI = 147. The FT-IR spectrum of starting material, film on NaCl, *ν* cm^−1^: 3008 m, 2953 sh, 2923 vs, 2854 s, 1746 vs, 1654 w, 1465 m, 1377 m, 1240 m, 1163 m-s, 1099 m, 721 m, whereas its oxirane product had an FT-IR cm^−1^: 2926 vs, 2854 s, 1743 vs, 1460 m-s, 1379 m, 1237 m, 1168 m-s, 1102 m, 826 w-m, 722 w-m.

#### 3.1.15. Synthesis of Formyl Ester from Pennycress Oxirane

Epoxy pennycress oil (299.3 g, 312 mmol) and toluene (200 mL) were placed in a 1,000 mL 2-necked RBF fitted with a magnetic stirrer, a Dean Stark trap, and a reflux condenser. Formic acid (99 + %, 172.8 g) was added to the mixture which was then heated to gentle reflux. As with the soybean epoxide above, the reaction progressed and condensed water accumulated in the trap. The reaction was also monitored by sampling the mixture for diminution of the 825–845 cm^−1^ band of the starting oxirane. After 1.5 h, the FT-IR spectrum of a sample indicated the complete absence of the IR band of the epoxide (820–840 cm^−1^). The heat source was removed and the reaction mixture allowed to cool to RT. The solution was poured into stirring saturated NaHCO_3_ solution and extracted with ethyl acetate (150 mL). The aqueous layer was further extracted with more ethyl acetate (100 mL × 2) and the combined organic phases dried over Na_2_SO_4_ and concentrated under reduced pressure at 57°C. The yield was 362.2 g (87.5%); d^24^ = 1.096 g·mL^−1^; FT-IR spectrum (film on NaCl) cm^−1^: 2927 vs, 2854 vs, and 1729 vs (second derivative had two bands 1750 and 1727 cm^−1^), 1463, 1381, 1244, 1176, 1107, 1023, and 718.

## 4. Results and Discussion

**Figure 1:**
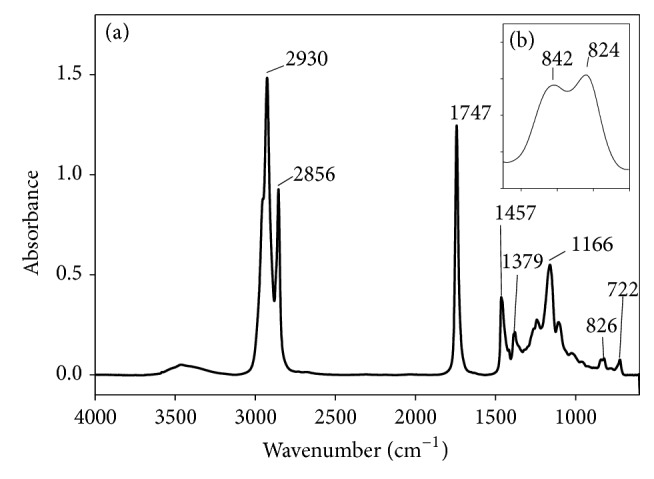
(a) FT-IR spectrum of the polyoxirane of milkweed oil; (b) epoxy absorption bands.

**Figure 2:**
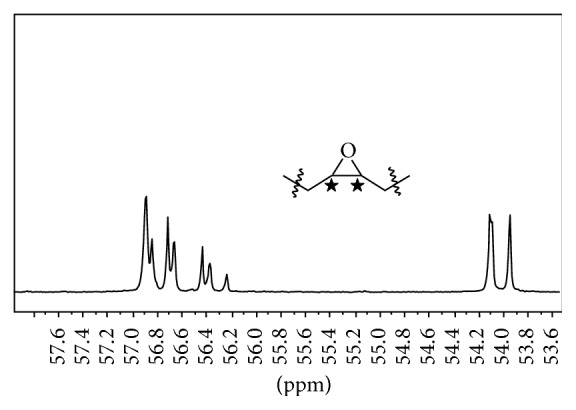
^13^CNMR spectral region of milkweed polyoxirane.

**Figure 3:**
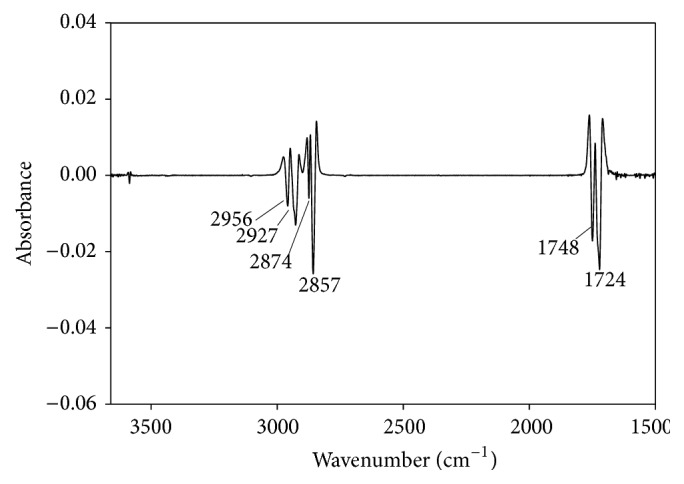
2nd derivative of the FT-IR spectrum of milkweed polyformyl ester.

**Figure 4:**
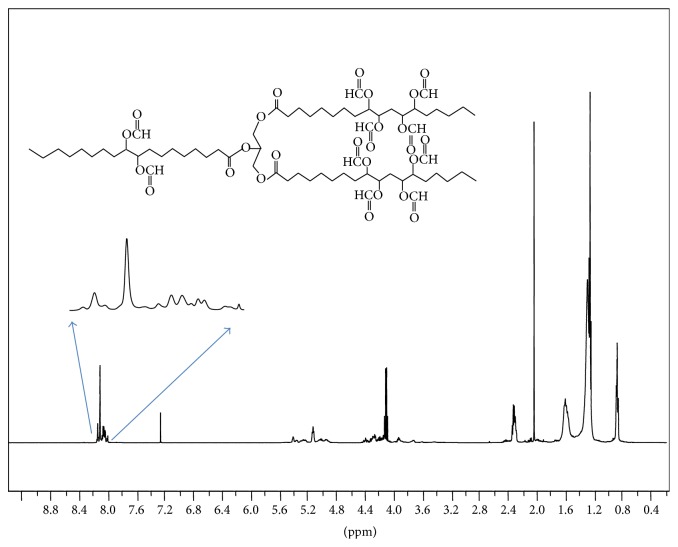
^1^HNMR spectrum of the polyformyl ester of milkweed oil. Insert is expanded region of methine frequencies (8.00–8.20 ppm).

**Figure 5:**
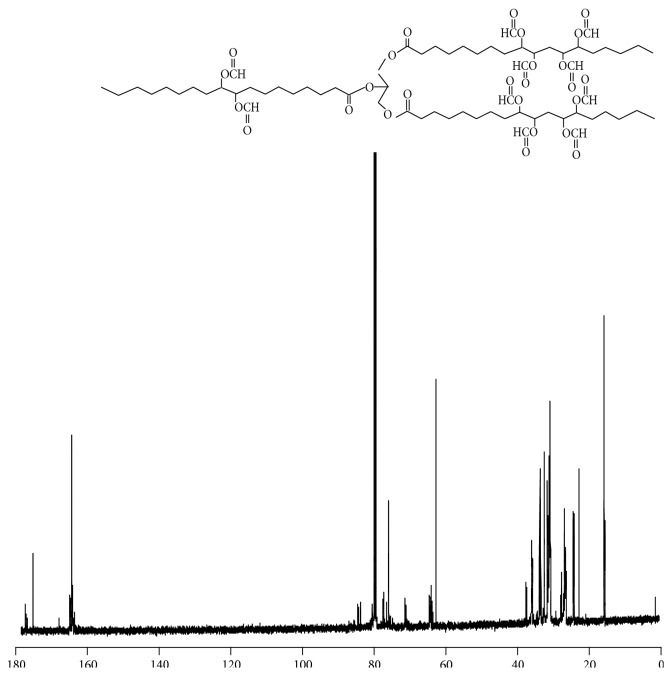
^13^CNMR spectrum of the polyformyl ester of milkweed oil.

**Figure 6:**
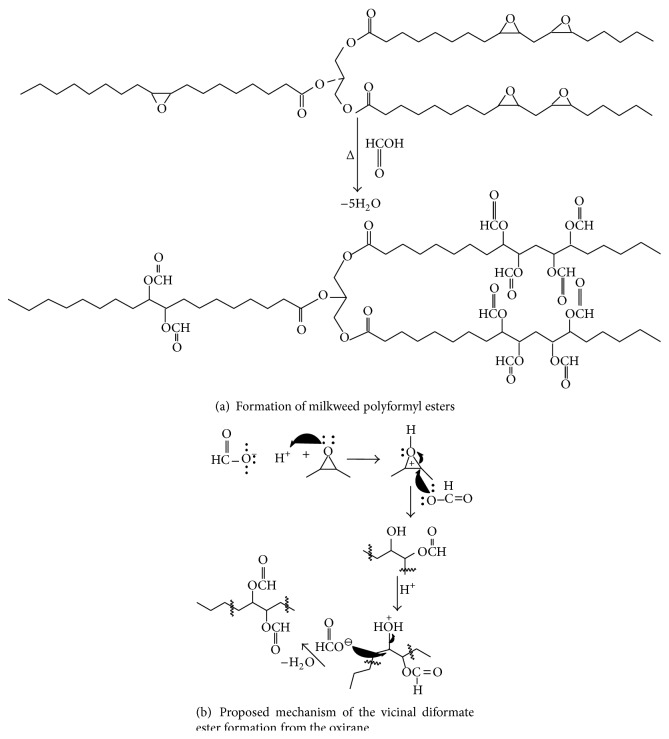
(a) Schematic of formation of polyformyl ester of milkweed oil from the oxirane; (b) proposed mechanism of the vicinal diformyl ester formation from the oxirane.

In an earlier study of acylation of polyhydroxylated milkweed oil to generate polyesters of useful lubricity characteristics [15] we had employed acid anhydrides (C2–C5). The data obtained then suggested that shorter chain substituent groups on the triglyceride acyl chains give more effective lubricating behavior than longer chain derivatives. By inference we decided to explore the effect of the shortest alkyl chain length analogue, that is, the formyl ester of the triglyceride* vis-à-vis* the longer chain substituent groups. But formic anhydride is a scarce reagent; hence we opted to approach the formyl ester of the triglyceride via the oxirane rather than the polyhydroxyl pathway. The principal diagnostic feature of the oxirane starting material for the formyl ester synthesis is the 820–845 cm^−1^ doublet in the FT-IR spectrum, Figure 1. This is the epoxy -C-O-C- asymmetric stretching mode of the three-membered ring. The corresponding ^13^CNMR resonances for the oxirane spectrum are the ten carbon resonance lines between 57 and 53.8 ppm, shown in Figure 2; this region of the spectrum is featureless in the starting triglyceride and in the subsequent formyl derivative. Conceptually, from the characteristically low pH 2.3 of formic acid, one would expect a facile epoxy ring-opening with the generation of an alpha-hydroxyl formate with or without a strong acid catalyst. So we opted not to use any catalyst. What was actually observed in the reaction process under gentle reflux as the reaction progress was monitored by sampling using FT-IR spectroscopy was the nonappearance of the expected hydroxyl group absorbance in its characteristic 3500–3400 cm^−1^ region as the epoxy ring absorption (820–840 cm^−1^) diminished. The inference was a condensation step following the initial ring-opening reaction of the epoxide. To confirm this postulate, toluene was added to the reaction mixture in a subsequent ring-opening reaction in order to azeotrope off any condensed water formed into a Dean Stark trap. This was confirmed by the quantity of water collected in the trap during the reaction. Thus the resulting ring-opening of the oxirane was polyformylation giving a vicinal diformyl ester per oxirane unit of the substrate. The FT-IR spectrum of the product Figure 3 shows a strong carbonyl band whose 2nd derivative indicates the presence of two carbonyl types, that is, the parent triglyceride (-C=O) with a weaker carbonyl band intensity at 1751–1745 cm^−1^ and a very strong band at 1730–1728 cm^−1^ for the H-C=O ester. The latter observation derives from the mass effect of the ratio of methine carbonyls to that of the relatively fewer triglyceride carbonyl esters present. A moderately weak band generally observed around 3500 cm^−1^ is attributed to the overtone of the very intense carbonyl absorption band at 1728. The ^1^HNMR spectrum of the polyformyl ester of milkweed oil shows characteristic resonance frequencies of the formyl methine protons at 8.0–8.20 ppm, Figure 4. Expansion of this absorption region reveals some ten singlet resonances (insert) whereas the ^13^CNMR spectrum of the polyformyl ester shown in Figure 5 has methine carbonyls of the formate occurring upfield around 160-161 ppm relative to the parent triglyceride ester carbonyls that are farther downfield at around 170–174 ppm. Mechanistically, it can be inferred that the alpha-hydroxyl group of the formate ester putatively formed rapidly loses a H_2_O molecule following protonation by a second molecule of formic acid as shown in Figures 6(a) and 6(b). The conjugate base (formate anion) then displaces the conjugate acid (-OH_2_)^+^ in a presumably concerted reaction step, thus giving rise to the vicinal diformyl ester per oxirane unit of the starting material. The reaction is a general one for vegetable oil oxiranes as both soybean and pennycress oil oxiranes gave the analogous diformyl esters under these reaction conditions.

### 4.1. Physical Properties of Polyformyl Esters

Physical properties of polyformates of milkweed (MW), soybean (SOY), and pennycress (PC) oils are summarized in Table 1. Also included in Table 1 for comparison are similar data from the literature [14–18] for the epoxides which are the starting materials for the formates. The density data in Table 1 indicate that the formates are also heavier than water. Comparison of the three polyformate triglycerides shows their density at 24°C increasing in the order: SOY < MW ≤ PC. Closer examination of the density data in Table 1 shows that the epoxides are also heavier than water whereas the vegetable oils are lighter than water. The low density of the vegetable oils can be attributed in part to the* cis* double bonds in the fatty acid structures which make efficient packing of the liquid oils difficult. However, elimination of the double bond through epoxidation is not expected to eliminate all of the* cis* configurations in the molecule. However, the increase in density of the epoxidized oil can be attributed in part to increase in the mass of the molecule (due to -O- insertion) without significant corresponding increase in volume and partly from the significant change in bond angle from the sp^2^ hybrid of the C=C to an sp^3^ hybridization which results in better packing of the chains in spite of the strain imposed by the three-membered rings. The data in Table 1 show that formate generation from the epoxide resulted in further increase of density for PC and MW, but a slight decrease for SOY. The additional density increases observed in PC and MW formate can be rationalized in terms of better packing (lower volume) of the molecules due to the expected elimination of all* cis* configurations.

Table 1 also compares the surface tension (ST) of the polyformyl esters at room temperature. The data show that, within one standard deviation, SOY and MW formates have identical ST values, which were slightly lower than the value for PC. The similar ST values for SOY and MW can be rationalized in terms of similarity in their fatty acid composition [21] (Table 2). Most (99%) of the fatty acids in MW and SOY comprise mixtures of C16 to C18 fatty acids of similar degrees of unsaturation, *T*_unsat_, defined as follows:

(1) $$\begin{array}{c} T_{unsat}=\Sigma \left(\;i*w_{i}\;\right), \end{array}$$

where *w*_*i*_ is weight percent of fatty acid with *i* double bonds; *i* = 1, 2, 3.

As shown in Table 2, *T*_unsat_ for SOY and MW are 153.6 and 154.8, respectively. What this means is that these two polyformate oils comprise mixtures of triglycerides whose chemical structures have about equal numbers of formate groups placed in about similar locations on fatty acids of similar chain length distribution. On the other hand, Table 2 shows that PC has a totally different fatty acid composition [14] and a much lower *T*_unsat_. In PC, nearly half (~48%) of the fatty acids have chain length of C20 or higher, whereas the content of such fatty acids in SOY and MW is negligible (<1%). PC also has a *T*_unsat_ value of 143, which is about 10% lower than the values in SOY and MW. It appears that a combination of high composition of long chain fatty acids and lower *T*_unsat_ value has resulted in a slightly higher ST for PC than for SOY or MW. This observation is contrary to expectation based on polarity argument. According to polarity reasoning, higher ST corresponds to a higher degree of polarity of the molecule, which should increase with increasing *T*_unsat_, hence increasing the number of formate groups per molecule. The fact that molecules with higher *T*_unsat_ have lower ST than a molecule with lower *T*_unsat_ means a different mechanism is responsible for the observed ST values. One possible explanation is that the long chain fatty acid might induce generation of a lipophilic region, similar to that observed in surfactants [22], thereby forcing the formate groups to the surface and causing an increase in surface tension of the oil.

*Viscosities*. The kinematic viscosities (kinVisc) at 40 and 100°C and viscosity indices (VI) of these formates and their precursor oxiranes and the native vegetable oils are compared in Table 1. The viscosity of SOY formate at both temperatures was 3- to 4-fold higher than the values for MW and PC formates. It is not clear why SOY formate should be so much more viscous than MW formate, considering that these two oils have reasonably similar fatty acid composition and *T*_unsat_. Based on molecular weight considerations, it is possible to rationalize higher viscosity for SOY formate relative to PC formate. The latter has lower *T*_unsat_ and, hence, will have a lower molecular weight than the former. This reasoning, unfortunately, breaks down when comparing the viscosities of MW and PC formates. Molecular weight argument predicts higher viscosity for MW formate relative to PC formate, but the opposite trend was observed. However, further examination of Table 1 shows that the molecular weight argument correctly predicts most of the viscosity data as follows: (a) the viscosity of SOY and MW epoxides, at 40 and 100°C, were close to each other but much higher than that of PC epoxide. This is consistent with a higher molecular weight of SOY and MW epoxides, due to higher *T*_unsat_, values, than that of PC epoxide; (b) the viscosities of SOY and MW vegetable oils at 40 and 100°C were similar but lower than that of PC vegetable oil. This is consistent with the higher molecular weight of PC because its fatty acid profile is almost half C16–C18 and half C20–C24, whereas SOY and MW comprise almost 100% C16–C18 fatty acids. (c) Comparison of the viscosities at 40 and 100°C of vegetable oils, epoxides, and formates for each type of oil (SOY, MW, and PC) shows viscosity increasing in the order: veg. oil < epoxide < formate. This trend corresponds to increasing molecular weight as a result of epoxidation and formylation.

Finally, Table 1 compares the viscosity index (VI) of formates and the precursor oils. The VI is a measure of the effect of temperature on viscosity. In general, viscosity decreases with increasing temperature and lubricants which display a smaller viscosity decrease with increasing temperature have higher VI values and are preferred [23]. The data in Table 1 shows that the unmodified vegetable oils have high VI properties, with values close to 200 and higher. This confirms a widely recognized superiority of vegetable oils over petroleum-based oils whose VI is generally below 150 [21, 23]. The data in Table 1 also shows that epoxidation of the vegetable oil results in a sharp decrease of VI in each vegetable oil. This phenomenon, where a chemical modification causes a reduction in VI of vegetable oils, has also been observed by others [17, 19]. A possible explanation of this observation could be expansion of the structural variation in the vegetable oil mixture due to chemical modification, resulting in components with wide differences in their viscosity and VI properties. The data in Table 1 also shows that conversion of the epoxides into formates caused an increase in VI for all three vegetable oils. It means, using the argument mentioned above, that conversion of the epoxide into the formate has narrowed the structural variation in the mixture. One possible mechanism given earlier for this observation is that conversion of the* cis* epoxides to the vicinal diformates which are* trans* in orientation (Figure 6(b)) significantly reduced the structural variation in the mixture.

### 4.2. Oxidative Stability

Stability to oxidation of the polyformate vegetable oils was evaluated using pressurized differential scanning calorimetry (PDSC). Samples were tested in a cell pressurized with pure oxygen under dynamic conditions, that is, with positive oxygen flow rate of 100 ± 10 mL/min. The method provides the oxidation onset (OT) and peak (PT) temperatures. OT and PT correspond to the temperature when oxidation begins and produces maximum heat flow, respectively. Higher OT and PT correspond to improved oxidation stability.  Table 3 compares the OT and PT values for the soy, MW, and PC formates. Also included in Table 3 are available similar data on the epoxide and vegetable oil precursors.

Examination of Table 3 shows the OT and PT for the formates decreasing in the order: MW > SOY > PC. However, close examination of the OT and PT data shows that these values for the three formates were within ±5-6°C from each other. Thus it can be assumed that the three formates will have similar oxidation stabilities.

Examination of Table 3 shows a larger difference in OT and PT (ΔOT and ΔPT, resp.) when comparing data for SOY veg. oil vs. SOY formate. Both OT and PT increased significantly when the veg oil was converted to the formate via epoxidation. This increase in OT and PT corresponds to a real improvement in oxidation stability of SOY due to conversion of the double bonds to formate esters. Poor oxidation stability of vegetable oils is attributed to reactive (allylic and bisallylic) protons caused by unsaturation in the triglyceride structures [23, 24]. A much more complex picture is observed when comparing OT and PT values for epoxides vs. formate esters of MW (Table 3). Conversion of MW epoxide to formate reduced OT but increased PT. This contradictory result in ΔOT and ΔPT is an indication that no change in oxidation stability has occurred in the manipulation of epoxide to formate chemical transformation. This is expected since no allylic or bisallylic protons are eliminated or generated as a result of the reaction.

### 4.3. Tribological Properties

Tribological investigations of formate derivatives of SOY, MW, and PC oils were conducted using a 4-ball tribometer. In the 4-ball tribometer, test is conducted for a specified time between three stationary bottom balls immersed in a lubricant at a specified temperature and a top ball rotating at a specified speed and pressed against the three bottom balls with a specified load. Two types of 4-ball test were conducted: antiwear (AW) (ASTM D 4172) [11, 12] and extreme pressure (EP) (ASTM D 2783) [13]. In the AW procedure, tests were conducted for 60 min in the sample lubricant at 75°C at 1200 rpm and an applied load of 392 N. In the EP test, a series of 10 s tests were conducted at room temperature with progressively increasing load, and 1760 rpm until welding of the 4 balls occurred. In the AW test, the average coefficient of friction (COF) and wear scar diameter (WSD) on the three bottom balls are reported. In the EP test the weld point (WP), that is, the load which caused the four balls to weld, is reported.

The 4-ball tribological test results for the formates and available values for SOY and PC veg. oils are summarized in Table 4. Examination of the AW results shows that the three formates have similar COF, which was in the range of 0.05 to 0.06. However, the three formates did not show similar WSD but displayed values that increased in the order: MW < PC < SBO. Based on the fatty acid composition of the oils given in Table 2, one would have expected similar WSD for SOY and MW which are different from PC. The data, however, indicates that the WSD for PC is closer to SOY than the WSD of SOY was to MW which exhibited the lowest wear. It is not clear why WSD results do not follow the trend of the fatty acid composition of the oils.

Further examination of the AW results in Table 4 indicates substantial reductions in both COF and WSD when the MW, SOY, and PC vegetable oils were converted to the corresponding formyl esters of the triglycerides. These changes in both COF and WSD can be attributed to changes in viscosity due to chemical modification of the vegetable oils to the corresponding formates. Examination of the EP results in Table 4 indicates that the three formates had similar WP values which were in the range 140 to 160 kgf. This result is expected since the three formates comprise similar elements (C, H, O) in their structures and will produce similar tribofilms (carbides, oxides, etc.) during the tribochemical process of the EP test [25].

## Figures and Tables

**Table 1:** Some physical properties of polyformate esters of vegetable oils^(a)^.

		SOY	MW	PC
Density at 23–25°C (g/mL)	Veg. oil	0.919 at 25°C^(b)^	<1 at 23°C (0.913)^(c)^	0.896 at 25°C
	Epoxide	1.111 at 24°C^(d)^	1.05 at 23°C^(c)^	1.023 at 24°C
	Formate	1.041 at 24°C	1.09 at 24°C	1.096 at 24°C

Surface tension at RT, dyn/cm	Formate	34.3 ± 0.1	34.5 ± 0.2	35.4 ± 0.2

kinVisc at 40°C (mm^2^/s)	Veg. oil	32.7^(e)^	33.8^(c)^	40.97^(f)^
	Epoxide	170.9^(d)^	164.4^(c)^	136
	Formate	1493.9^(c)^	388.3^(c)^	595.7^(c)^

kinVisc at 100°C (mm^2^/s)	Veg. oil	7.5^(e)^	7.3^(c)^	9.39^(f)^
	Epoxide	20.4^(d)^	19.22^(c)^	18.00
	Formate	112.66	44.6	55.4

Viscosity Index	Veg. oil	209^(e)^	190	224^(f)^
	Epoxide	136^(d)^	133^(c)^	147
	Formate	170	172^(c)^	157

(a) All data from this work unless otherwise noted.

(b) at 25°C [16].

(c) [15].

(d) [17].

(e) [18].

(f) Data for crude pennycress from [14].

**Table 2:** Fatty acid composition of vegetable oils (% w/w).

Triglyceride	SOY^(a)^	Milkweed^(b)^	Pennycress^(c)^
C14:0	0.1	—	0.1
C16:0	10.6	5.7	3.1
C16:1/C16:2	0.1	9.6	0.2
C18:0	4.0	2.5	0.5
C18:1	23.2	31.0	12.6
C18:2	53.7	50.5	22.4
C18:3	7.6	1.2	11.8
C20:0	0.3	—	0.3
C20:1	—	—	8.6
C20:2	—	—	1.6
C22:0	0.3	—	0.6
C22:1	—	—	32.8
C22:2	—	—	0.7
C22:3	—	—	0.3
C24:1	—	—	2.9
Tunsat^(d)^	153.6	154.8	143

(a) Data for RBD soybean oil from [19].

(b) Data for crude milkweed from [20].

(c) Data for crude pennycress from [14].

(d) Tunsat (total unsaturates) = Σ(*i∗w*_*i*_), where *w*_*i*_ is weight percent of fatty acid with *i* double bonds; *i* = 1, 2, 3.

**Table 3:** Oxidation stability of vegetable oils, epoxides, and formate esters^(a)^.

		SOY	MW	PC
PDSC – OT (°C)	Veg. oil	162.0 ± 0.6		
	Epoxide		194.5 ± 0.3	
	Formate	179.1 ± 0.1	183.8 ± 0.5	172.7 ± 3.8

PDSC– PT (°C)	Veg. oil	171.3 ± 1.1		
	Epoxide		211.3 ± 0.5	
	Formate	217.8 ± 2.4	224.7 ± 1.5	215.5 ± 1.1

(a) All data from this work unless noted.

**Table 4:** Tribological properties of vegetable oils, epoxides, and formate esters^(a)^.

		SOY	MW	PC
4-ball AW - COF	Veg. oil	0.08^(b)^		0.07
	Epoxide			
	Formate	0.05 ± 0.01	0.06 ± 0.00	0.05 ± 0.00

4-ball AW – WSD (mm)	Veg. oil	0.70^(b)^		0.70
	Epoxide			
	Formate	0.60 ± 0.05	0.38 ± 0.01	0.52 ± 0.00

4-ball EP-WP (kgf)	Formate	160	140	160

(a) All data from this work unless noted.

(b) [19].
